# Manifestation of a Second Dirac Surface State and Bulk Bands in THz Radiation from Topological Insulators

**DOI:** 10.1038/srep14128

**Published:** 2015-09-15

**Authors:** Chien-Ming Tu, Tien-Tien Yeh, Wen-Yen Tzeng, Yi-Ru Chen, Hsueh-Ju Chen, Shin-An Ku, Chih-Wei Luo, Jiunn-Yuan Lin, Kaung-Hsiung Wu, Jenh-Yih Juang, Takayoshi Kobayashi, Cheng-Maw Cheng, Ku-Ding Tsuei, Helmuth Berger, Raman Sankar, Fang-Cheng Chou

**Affiliations:** 1 Department of Electrophysics, National Chiao Tung University, Hsinchu, Taiwan 300, R.O.C.; 2 Department of Electrical and Computer Engineering, National Chiao Tung University, Hsinchu, Taiwan 300, R.O.C.; 3 Taiwan Consortium of Emergent Crystalline Materials, Ministry of Science and Technology, Taipei 10601, Taiwan, R.O.C.; 4 Institute of Physics, National Chiao Tung University, Hsinchu, Taiwan 300, R.O.C.; 5 Advanced Ultrafast Laser Research Center and Department of Engineering Science, The University of Electro-Communications, Chofugaoka 1-5-1, Chofu, Tokyo 182-8585, Japan; 6 National Synchrotron Radiation Research Center, Hsinchu, Taiwan 300, R.O.C.; 7 Institut de Physique des Nanostructures, Ecole Polytechnique Fédérale de Lausanne (EPFL), CH-1015 Lausanne, Switzerland; 8 Center for Condensed Matter Sciences, National Taiwan University, Taipei 106, Taiwan, R.O.C.

## Abstract

Topological insulators (TIs) are interesting quantum matters that have a narrow bandgap for bulk and a Dirac-cone-like conducting surface state (SS). The recent discovered second Dirac surface state (SS) and bulk bands (BBs) located ~1.5 eV above the first SS are important for optical coupling in TIs. Here, we report on the time-domain measurements of THz radiation generated from TIs n-type Cu_0.02_Bi_2_Se_3_ and p-type Bi_2_Te_3_ single crystals by ultrafast optical pulse excitation. The observed polarity-reversal of the THz pulse originated from transient current is unusual, and cannot be reconciled with the photo-Dember effect. The second SS and BBs are found to be indispensable for the explanation of the unusual phenomenon. Thanks to the existence of the second SS and BBs, TIs manifest an effective wide band gap in THz generation. The present study demonstrates that time-domain THz spectroscopy provide rich information of the optical coupling and the electronic structure of TIs.

A new phase of quantum matters called topological insulators (TIs) are theoretically predicted and experimentally observed recently, and their band structures are composed of insulating bulk bands and a conducting surface state (SS). The SS results from a strong spin-orbital interaction and band inversion^1^,^2^ and exhibits novel properties, such as the protection by time-reversal symmetry against backscattering from nonmagnetic impurities^3^,^4^,^5^ and spin-polarized current^6^,^7^,^8^. These properties hint at potential applications in spintronic devices and quantum computations. Dirac fermions in TIs have been extensively studied using angle-resolved photoemission spectroscopy (ARPES)^9^,^10^,^11^ or scanning tunneling microscopy (STM)^12^,^13^,^14^,^15^. Optical techniques, such as surface second harmonics generation (SHG), have provided an alternative contact-free detection for Dirac Fermion^16^,^17^, and ultrafast optical techniques also have been used to study the characteristics of TIs^18^,^19^,^20^,^21^,^22^. Very recently, an unoccupied 2nd SS and bulk bands (BBs) which are located above the 1st SS were predicted^23^ and observed in the material family of Bi_2_Te_x_Se_3-x_ using ARPES and two-photon photoemission measurements^24^,^25^. The 2nd SS also originates from the strong spin-orbital coupling and is expected to keep the same topological and spin characteristics as the 1st SS^23^,^24^. However, the role of the 2nd SS and BBs on the interactions between TIs and photons has been much less investigated. Further explorations on the relevant subjects are indispensable to the development of TI optical devices.

This study reports the time domain measurements of THz radiation generated from the surface and bulk of n-type Cu_0.02_Bi_2_Se_3_ and p-type Bi_2_Te_3_ single crystals. Two different mechanisms of THz generation: optical rectification (OR) and transient current, have been identified by different polarization configurations of both optical excitation and THz radiation. With this time-domain technique, the 2nd SS and BBs manifest themselves in the spectrum, and prove vital in generating the THz radiation through a transient drift current.

## Results

### P-polarized THz radiation from TIs

**Figure 1:**
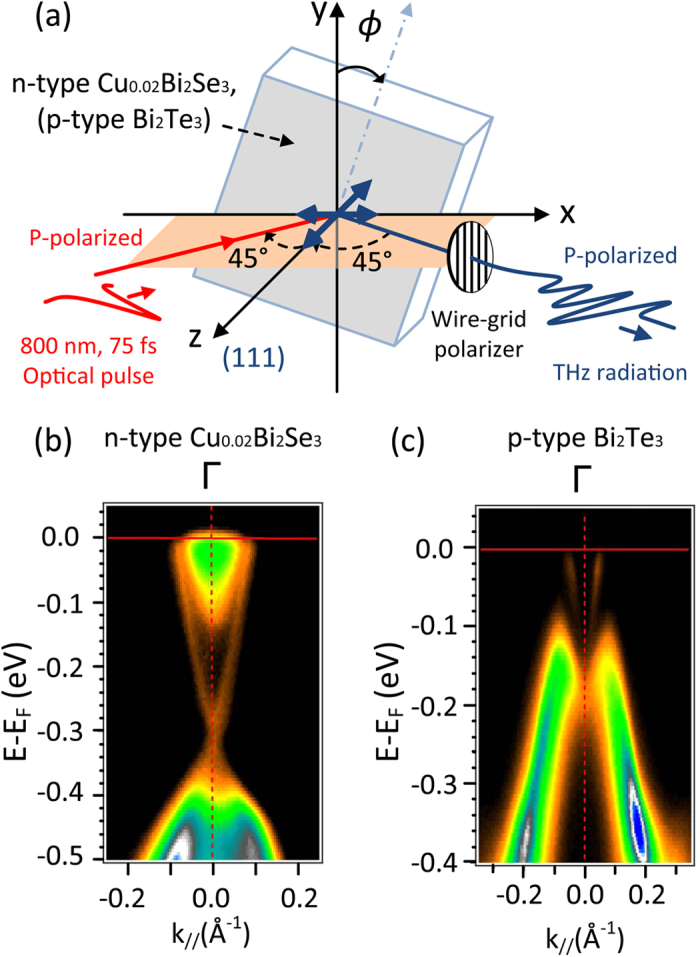
(**a**) Schematic for THz generation. P-polarized optical pulses irradiated the (111) surface of TIs and P-polarized THz radiation was detected after a wire-grid polarizer. The thick arrows denote in-plane and out-of-plane electric fields. (**b**,**c**) ARPES images of n-type Cu_0.02_Bi_2_Se_3_ and p-type Bi_2_Te_3_.

**Figure 2:**
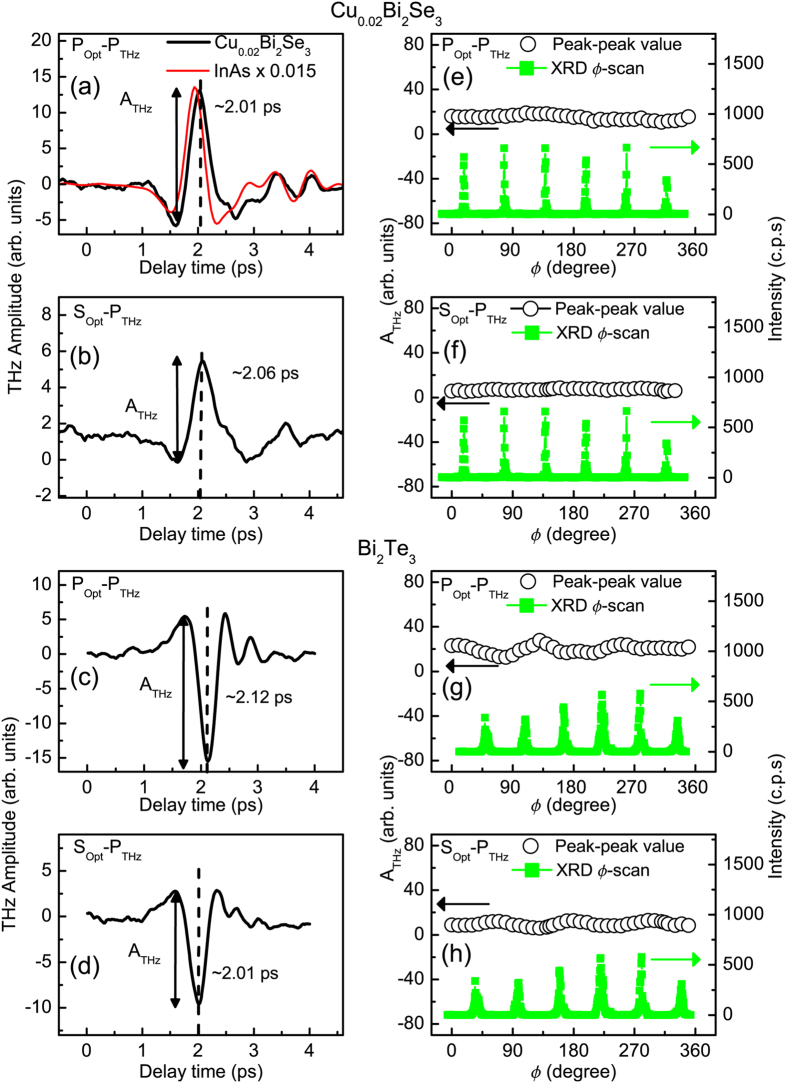
(**a**–**d**) Time-domain P-polarized THz waveforms radiated from the (111) surface of n-type Cu_0.02_Bi_2_Se_3_ and p-type Bi_2_Te_3_ by P- and S-polarized optical pulse excitation. In (**a**), the thin-red line represents the 0.015-times smaller P-polarized THz wave that is generated from an n-type InAs under the same conditions. In both P_Opt_-P_THz_ and S_Opt_-P_THz_ configurations, the polarities of the P-polarized THz waveforms from p-type Bi_2_Te_3_ (**c**,**d**) are in the reverse of that from n-type Cu_0.02_Bi_2_Se_3_ (**a**,**b**). A_THz_ is the peak-to-peak value of the THz waveform. (**e**–**h**): the plots of A_THz_ for the P-polarized THz waveforms as a function of azimuthal angles (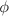-scan) of Cu_0.02_Bi_2_Se_3_ (**a**,**b**) and Bi_2_Te_3_ (**c**,**d**) single crystals (black). The corresponding XRD 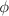-scans for the (1 1 15) peak for both single crystals are also shown in (**e**–**h**) (green).

The experimental setup was a reflective type, where the optical pulse illuminated the (111) surfaces of the TI crystals at an incident angle of 45°, to generate THz radiation, as shown in Fig. 1(a). Two types of TI single crystals were used in this study: Cu-doped n-type Bi_2_Se_3_ (Cu_0.02_Bi_2_Se_3_) with a carrier concentration of 3.66 × 10^18^ cm^−3^ and p-type Bi_2_Te_3_ with a carrier concentration of 1.32 × 10^19^ cm^−3^. Both were determined using the Hall measurements and the ARPES images of the employed single crystals are shown in Fig. 1(b,c). Figure 2(a,b) show the typical time domain THz waveforms with several cycles for the n-type TIs. In these two cases, the P- and S-polarized optical pulse illuminated the surface of an n-type Cu_0.02_Bi_2_Se_3_ single crystal and a wire-grid polarizer was set to detect P-polarized THz waves (denoted by P_Opt_-P_THz_ and S_Opt_-P_THz_), respectively. Figure 2(e) shows the dependence of the azimuthal angle (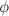-scan) of the peak-to-peak value of the THz pulse (A_THz_) from the (111) surface of Cu_0.02_Bi_2_Se_3_ in P_Opt_-P_THz_ configuration and a corresponding x-ray diffraction (XRD) 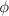-scan of the (1 1 15) peak of TI crystals. The result in S_Opt_-P_THz_ configuration is shown in Fig. 2(f). In these two configurations, the 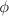-dependent THz peak-to-peak value (THz 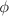-scan) does not show a significant three-fold symmetry associated with that of the crystalline structure of Cu_0.02_Bi_2_Se_3_. Because the P-polarized THz radiation contains both in-plane and out-of-plane electric-field components, the THz 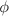-scan results show that the P-polarized THz radiation is dominated by the out-of-plane component from the bulk of the TIs, as shown in Fig. 1(a). Obviously, the THz waveforms from Cu_0.02_Bi_2_Se_3_ are similar to that from InAs (thin-red line in Fig. 2(a)) but the amplitude of the P-polarized THz pulse from Cu_0.02_Bi_2_Se_3_ is much smaller than that from InAs under the same conditions.

Figure 2(c,d) show the typical time-domain THz waveforms from a p-type Bi_2_Te_3_ single crystal in the configurations of P_Opt_-P_THz_ and S_Opt_-P_THz_, respectively. The shape and amplitude are almost the same as those for the n-type Cu_0.02_Bi_2_Se_3_ in Fig. 2(a,b). The THz 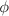-scans in Fig. 2(g,h) do not show a significant three-fold symmetry associated with the crystalline structure of Bi_2_Te_3_, either. Nevertheless, the polarities of the P-polarized THz pulse from p-type Bi_2_Te_3_ are opposite to those from the n-type Cu_0.02_Bi_2_Se_3_. This intriguing contrast provides a revealing insight into TIs’ optical coupling and novel electronic structure as we shall show later.

### S-polarized THz radiation from TIs

**Figure 3:**
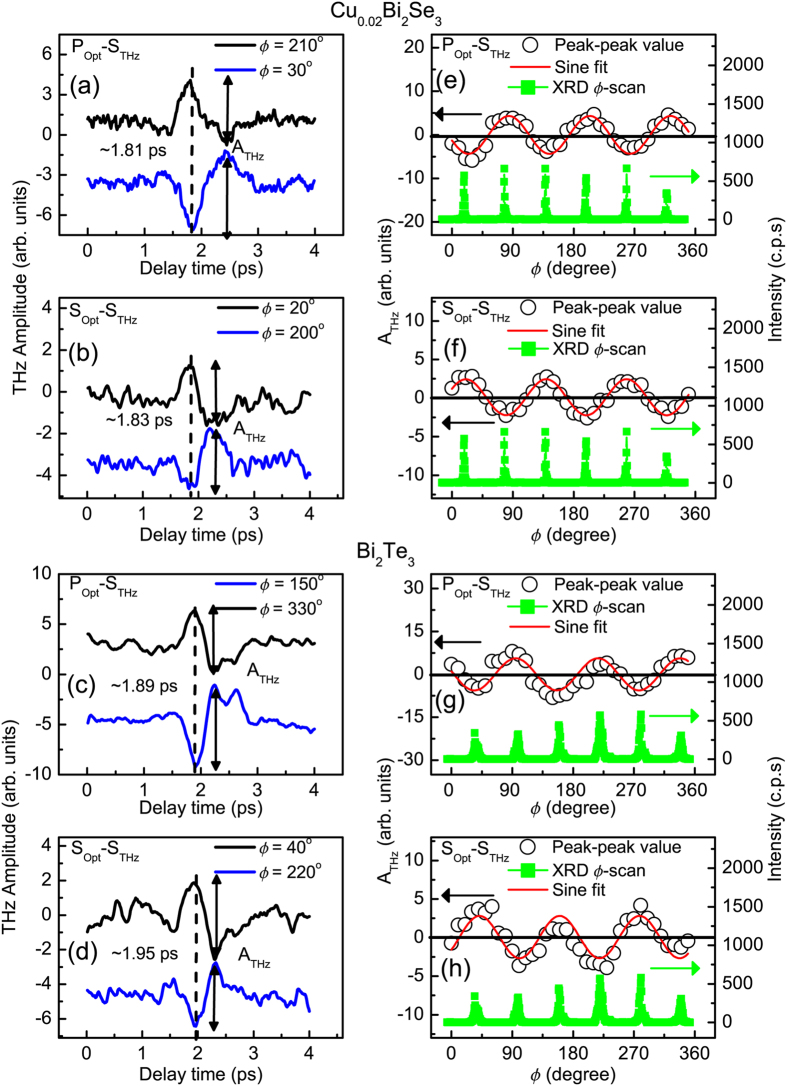
(**a**–**d**) Time-domain S-polarized THz waveforms radiated from the (111) surface of n-type Cu_0.02_Bi_2_Se_3_ and p-type Bi_2_Te_3_ by P- and S-polarized optical pulse excitation. In all polarization configurations, the polarities of S-polarized THz waveforms are reversed when the samples are rotated 180° along the surface normal. A_THz_ is the peak-to-peak value of the THz waveform. (**e**–**h**): the plots of A_THz_ for the S-polarized THz waveforms as a function of azimuthal angles (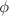-scan) of Cu_0.02_Bi_2_Se_3_ (**a**,**b**) and Bi_2_Te_3_ (**c**,**d**) single crystals (black). Obviously, the THz 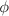-scans for both single crystals match the XRD 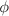-scans for the (1 1 15) peak (green).

In the experiments of the P_Opt_-P_THz_ and S_Opt_-P_THz_ configurations, the in-plane contribution to THz radiation cannot be separated. To obtain the in-plane contribution to THz radiation, the wire-grid polarizer and the EO sampling setup were rotated to detect the S-polarized THz radiation under P- and S-polarized optical excitation (P_Opt_-S_THz_ and S_Opt_-S_THz_). Figure 3(a) shows the time-domain THz waveforms from the (111) surface of n-type Cu_0.02_Bi_2_Se_3_ in the P_Opt_-S_THz_ configuration. The results in S_Opt_-S_THz_ configuration is also shown in Fig. 3(b). Although the signal-to-noise ratio is low (about 2 to 3), it still can be observed that the polarity of the THz waveform is reversed if the azimuth angle of samples is rotated by 180°. The similar results of p-type Bi_2_Te_3_ are shown in Fig. 3(c,d). The origins of S-polarized THz radiation shall be discussed later.

## Discussion

For THz generation from common semiconductors, there are three possible mechanisms: (1) optical rectification (OR), (2) the photo-Dember effect and (3) the surface field effect^26^. In the experimental results, the P-polarized THz radiation from both Cu_0.02_Bi_2_Se_3_ and Bi_2_Te_3_ do not show a significant three-fold symmetry that is related to the crystalline structure, and this excludes OR as the mechanism for P-polarized THz radiation from TIs. However, both the photo-Dember effect and the surface field effect are associated with transient current from photocarriers that are excited by an optical pump pulse inside the material. The far-field electrical field 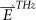 for THz radiation is described by


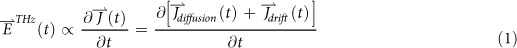


where 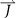 is the transient current created by an optical pulse, and it is composed of diffusion current 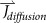 and drift current 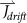. The amplitude of 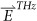 is proportional to the time-derivative of 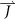. In general, the direction of 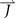 is normal to the surface of the material, and it contributes to the P-polarized THz radiation if the pump beam is obliquely incident. The polarity of the THz radiation also depends on the direction of 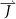, whether it is inward to or outward from the surface of the material. The photo-Dember effect is a result of the concentration gradient of the photoexcited carriers near the semiconductor surface. Because there is a large difference between photoexcited electrons and holes in the diffusion coefficient and the mobility, photoexcited electrons in the bulk of a material diffuse much faster than holes, which causes the diffusion current 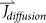 to be the main component of 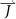 in Eq. (1). Therefore, the photo-Dember effect is the dominant mechanism for THz generation from narrow bandgap semiconductors^26^, such as InAs (bandgap ∼ 0.36 eV) and InSb (bandgap ∼ 0.17 eV). In this case, the direction of 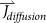 is always outward from the surface and the polarity of the THz pulses must be the same for both n- and p-type semiconductors. Although both n-type Cu_0.02_Bi_2_Se_3_ (bandgap ∼ 0.3 eV) and p-type Bi_2_Te_3_ (bandgap ∼ 0.15 eV) could be classified as narrow bandgap semiconductors, the photo-Dember effect cannot explain the reversed polarity of P-polarized THz radiation between n- and p-type TIs.

For wide bandgap semiconductors, such as GaAs (bandgap ∼ 1.43 eV), THz radiation can be explained by the surface field effect which is due to the acceleration of photoexcited carriers by the surface field near the semiconductor surface. The surface field is caused by band-bending due to Fermi-level pinning by the surface state^26^. The photoexcited electrons and holes are accelerated in opposite directions by the surface field to form a transient drift current 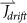. In general, the direction of the surface field in n-type semiconductors is opposite to that in p-type semiconductors. Therefore, the directions of the induced transient currents are opposite and the polarity of THz pulses from n-type semiconductors is also opposite to that from p-type semiconductors.

**Figure 4:**
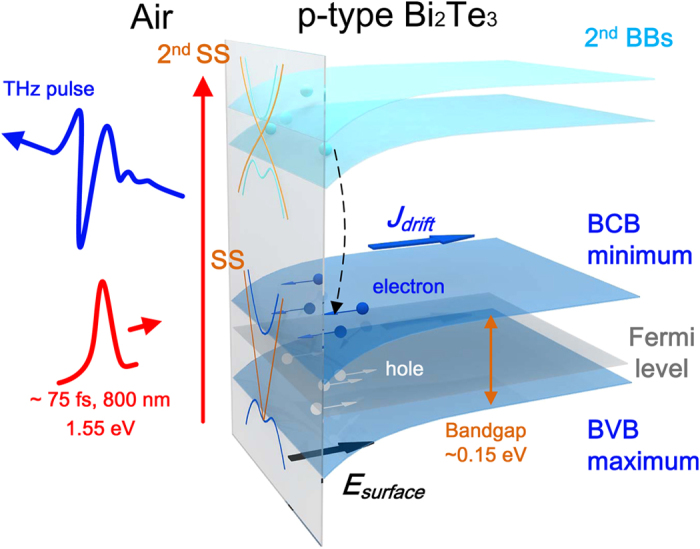
Schematic for the influence of the 2nd surface state (SS) and bulk bands (BBs) on the P-polarized THz generation from p-type Bi_2_Te_3_. BCB: bulk conduction band. BVB: bulk valance band. The 2nd SS and BBs would be responsible for the energy loss of the photoexcited electrons. Thus, the surface field 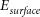 induced by band-bending also results in the drift current 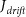 in the narrow bandgap materials.

For Bi_2_Se_3_ and Bi_2_Te_3_, surface band-bending has been observed by previous studies, using angle-resolved photoemission spectroscopy, scanning tunneling spectroscopy, and hard x-ray photoelectron spectroscopy^27^,^28^,^29^,^30^. Theoretical calculations also predict that the sign of band-bending could be affected by the type of doping: electron doping or hole doping^31^. Therefore, the polarity of P-polarized THz radiation from TIs is affected by the TIs’ doping. The experimental results indicate that 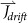 predominates over the photoexcited current inside p-type Bi_2_Te_3_, which further implies that the excess energy of the photoexcited carrier is too low to form a sufficiently strong photo-Dember field. However, from an energy point of view, the *excess energy* that the photoexcited carriers gain from the pumping photon energy is larger than that given by the surface field for narrow band semiconductors as in TIs. In this regard, 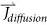 from the photo-Dember field should have been the dominant component of the photoexcited current^32^. This paradox can be beautifully resolved in the context of the 2nd SS and BBs in TIs. Very recently, using two-photon photoemission in ARPES measurements^24^,^25^, an unoccupied 2nd SS and BBs located 1.1 to 1.5 eV above the 1st SS were observed in the material family of Bi_2_Te_x_Se_3-x_. For Bi_2_Se_3_, the energy difference between the 2nd band (2nd SS and BBs) and 1st band (1st SS, bulk conduction band: BCB and bulk valance band: BVB) matches the photon energy of 1.55 eV. It has also been confirmed that direct transitions to the 2nd SS and BBs can be driven by 1.55 eV optical pulses in n-type samples^25^. This indicates the excess energy of the photoexcited carriers would be reduced dramatically. For Bi_2_Te_3_, the 2nd SS is closer to the 1st SS^24^ than that of Bi_2_Se_3_. For the p-type Bi_2_Te_3_ samples, however, the energy difference between the 2nd band (2nd SS and BBs) and the 1st band (1st SS, BCB and BVB) would still lead to sufficient energy loss of the photoexcited carriers. As shown in Fig. 4, to have a brief summary, *thanks to the existence of 2nd SS and BBs, TIs manifest an effective wide band gap in THz generation*. The 2nd SS and BBs could result in energy loss for these photoexcited carriers that generate THz radiation in TIs. Therefore, 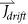, rather than 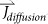, may predominate over the photoexcited current in TIs and produce THz pulses with opposite polarity, due to the doping dependent band-bending.

The band-to-band transitions may contribute to the absorption of pumping photon to cause the reduction of energy. In general, this phenomenon can be described by the Fermi Golden rule, which is a way to calculate the transition probability between the initial and final density of states. This indicates that the band-to-band transitions could be further predicted by the statuses of initial and final density of states. For both cases of n-type Cu_0.02_Bi_2_Se_3_ and p-type Bi_2_Te_3_, the Fermi surface locates in the 1st band and no carriers fill the 2nd band and higher energy bands. While the samples were pumped by a femtosecond laser pulse with photon energy of 1.55 eV, the transition would predominantly occur between the 1st and 2nd band at the initial stage according to the Fermi Golden rule. Even there are some photoexcited carriers on 2nd band after pumping, the band-to-band transitions from the 2nd band and higher energy bands can be neglected due to the femtosecond ultrashort pulse (<75 fs), which doesn’t provide enough time for the further absorption of photoexcited carriers on the 2nd band or higher energy bands. On the other hand, if we use a long pulse for pumping, the band-to-band transitions should be considered for the THz generation on TIs.

In the cases of S-polarized THz radiation from TIs, as shown in Fig. 3(a,b), although the signal-to-noise ratio of the measured THz waveforms is low (about 2 to 3), it still can be observed that the polarity of the THz waveform is reversed if the azimuth angle of samples is rotated by 180°. Further, the THz 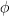-scans in Fig. 3(e,f) also show a three-fold symmetry, which is consistent with the surface crystalline structure of Cu_0.02_Bi_2_Se_3_. A similar symmetry was also recently observed in SHG signal using ultrafast optical pulse illumination in Bi_2_Se_3_^16^,^17^. For p-type Bi_2_Te_3_, the same phenomena are also observed, as shown in Fig. 3(c,d,g,h). Consequently, it is concluded that the OR is the main mechanism for S-polarized THz radiation from a TI surface. In general, the contribution of OR is not negligible in P-polarized THz radiation from the semiconductors, such as InAs and GaAs^26^. Because there is weak S-polarized THz radiation for Cu_0.02_Bi_2_Se_3_ and Bi_2_Te_3_, there is no significant modulation in the 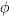-scan results for P-polarized THz radiation, as shown in Fig. 2(e–h).

Second-order nonlinear optical process is a simultaneous process in fs time scale and it means that THz pulse originated from OR is generated simultaneously with the incidence of optical pulse. On the other hand, it takes time for the photoexcited electrons, in the 2nd SS and BBs, to relax to the 1st SS or BCB^33^. This indicates that THz pulse originated from this complex process would lag behind that from OR. Comparing with the P-polarized THz pulses in Fig. 2(a–d), the main peak of the S-polarized THz pulses has a lead of ∼0.2 ps over TIs, as shown in Fig. 3(a–d). This value is closed to the relaxation time 0.5 ps, which is obtained by recent time-resolved ARPES measurement, for the photoexcited electrons from the 2nd SS and BBs relaxing to the 1st SS and BCB^33^. These results also demonstrate the influence of the 2nd SS and BBs on the P-polarized THz radiation from TIs. In this study, we cannot separate the contribution of the 2nd SS from that of 2nd BBs and the different-wavelength excitation may provide more insights into this unusual phenomenon. We also performed large photon-energy-excitation (central wavelength: 400 nm, E_photon_ = 3.1 eV) and the details are presented in Supplementary information.

In summary, we have characterized THz radiation from n-type Cu_0.02_Bi_2_Se_3_ and p-type Bi_2_Te_3_ single crystals using ultrafast optical pulse illumination. If an n-type Cu_0.02_Bi_2_Se_3_ single crystal is replaced by a p-type Bi_2_Te_3_ single crystal, there is a reversal in the polarity of THz radiation. These results suggest that the drift current 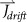 predominates over the transient current in these narrow bandgap materials. This notable phenomenon can be reconciled in the context of the second Dirac surface state and bulk bands. The S-polarized THz radiation results show a three-fold symmetry, which results from the second-order nonlinear optical effect of optical rectification. The present study demonstrates that the second Dirac surface state and bulk bands play the important roles in the optical coupling with the electronic structure of topological insulators.

## Methods

In the experiments, a mode-locked Ti: sapphire oscillator (XL 300, FEMTOLASERS) was used as the light source to generate a 300-nJ optical pulse train with a repetition rate 5.2 MHz and a central wavelength of 800 nm. The pulse duration is ∼75 fs. The output beam was split into two parts by an 80/20 beam splitter. The stronger one was used for THz generation and the weaker one for standard electro-optic sampling. A retro-reflector was mounted on a linear translation stage which was incorporated into the probe-beam path as an optical delay line. A half-wave plate and a cubic polarizer were used to vary the polarization of the optical pump beam. A wire-grid polarizer was used to detect the polarization of the generated THz radiation. Optical pump pulse illuminated the (111) surfaces of the TI crystals at an incident angle of 45° to generate THz radiation. The pulse energy of the pump beam was around 10 nJ and the spot size on the samples was about 280 μm in diameter. For 400-nm excitation, a nonlinear crystal slab, beta-BaB_2_O_4_ (BBO) of 0.5 mm in thickness, was used to generate 400-nm optical pulses by second harmonic generation. The THz radiation generated from the TI crystals was collimated by two off-axis parabolic mirrors and focused on a 1-mm-thick <110> ZnTe slab to detect THz radiation. All of the experiments were performed at room temperature and in a chamber filled with nitrogen gas to avoid THz-waveform distortion due to water vapor absorption.

## Additional Information

**How to cite this article**: Tu, C.-M. *et al.* Manifestation of a Second Dirac Surface State and Bulk Bands in THz Radiation from Topological Insulators. *Sci. Rep.* **5**, 14128; doi: 10.1038/srep14128 (2015).

## Supplementary Material

Supplementary Information

## References

[b1] Fu L., Kane C. L. & Mele E. J. Topological insulators in three dimensions. Phys. Rev. Lett. 98, 106803 (2007). 17358555 10.1103/PhysRevLett.98.106803

[b2] Qi X.-L., Hughes T. L. & Zhang S.-C. Topological field theory of time-reversal invariant insulators. Phys. Rev. B 78, 195424 (2008).

[b3] Moore J. E. The birth of topological insulators. Nature 464, 194–198 (2010). 20220837 10.1038/nature08916

[b4] Hasan M. Z. & Kane C. L. Colloquium: Topological insulators. Rev. Mod. Phys. 82, 3045 (2010).

[b5] Qi X.-L. & Zhang S.-C. The quantum spin Hall effect and topological insulators. Phys. Today 63, 33–38 (2010).

[b6] Raghu S., Chung S. B., Qi X.-L. & Zhang S.-C. Collective modes of a helical liquid. Phys. Rev. Lett. 104, 116401 (2010). 20366490 10.1103/PhysRevLett.104.116401

[b7] Hosur P. Circular photogalvanic effect on topological insulator surfaces: Berry-curvature-dependent response. Phys. Rev. B 83, 035309 (2011).

[b8] McIver J. W., Hsieh D., Steinberg H., Jarillo-Herrero P. & Gedik N. *et al.* Control over topological insulator photocurrents with light polarization. Nature Nano. 7, 96–100 (2012). 10.1038/nnano.2011.214 22138862

[b9] Hsieh D. *et al.* A topological Dirac insulator in a quantum spin Hall phase. Nature 452, 970–974 (2009). 18432240 10.1038/nature06843

[b10] Xia Y. *et al.* Observation of a large-gap topological-insulator class with a single Dirac cone on the surface. Nature Phys. 5, 398–402 (2009).

[b11] Chen Y. L. *et al.* Experimental realization of a three-dimensional topological insulator Bi_2_Te_3_. Science 325, 178–181 (2009). 19520912 10.1126/science.1173034

[b12] Zhang T. *et al.* Experimental demonstration of topological surface states protected by time-reversal symmetry. Phys. Rev. Lett. 103, 266803 (2009). 20366330 10.1103/PhysRevLett.103.266803

[b13] Roushan P. *et al.* Topological surface states protected from backscattering by chiral spin texture. Nature 460, 1106–1109 (2009). 19668187 10.1038/nature08308

[b14] Alpichshev Z. *et al.* STM Imaging of electronic waves on the surface of Bi_2_Te_3_: topologically protected surface states and hexagonal warping effects. Phys. Rev. Lett. 104, 016401 (2010). 20366373 10.1103/PhysRevLett.104.016401

[b15] Kim S. *et al.* Surface scattering via bulk continuum states in the 3D topological insulator Bi_2_Se_3_. Phys. Rev. Lett. 107, 056803 (2011). 21867088 10.1103/PhysRevLett.107.056803

[b16] Hsieh D. *et al.* Nonlinear optical probe of tunable surface electrons on a topological insulator. Phys. Rev. Lett. 106, 057401 (2011). 21405434 10.1103/PhysRevLett.106.057401

[b17] McIver J. W. *et al.* Theoretical and experimental study of second harmonic generation from the surface of the topological insulator Bi_2_Se_3_. Phys. Rev. B 86, 035327 (2012).

[b18] Kumar N. *et al.* Spatially resolved femtosecond pump-probe study of topological insulator Bi_2_Se_3_. Phys. Rev. B 83, 235306 (2011).

[b19] Luo C. W. *et al.* THz generation and detection on Dirac fermions in topological insulators. Adv. Optical Mater. 1, 804–808 (2013).

[b20] Luo C. W. *et al.* Snapshots of Dirac fermions near the Dirac point in topological insulators. Nano Lett. 13, 5797–5802 (2013). 24228733 10.1021/nl4021842

[b21] Sim S. *et al.* Ultrafast terahertz dynamics of hot Dirac-electron surface scattering in the topological insulator Bi_2_Se_3_. Phys. Rev. B 89, 165137 (2014).

[b22] Sim S. *et al.* Tunable Fano quantum-interference dynamics using a topological phase transition in (Bi_1-x_In_x_)_2_Se_3_. Phys. Rev. B 91, 235438 (2015).

[b23] Eremeev S. V., Silkin I. V., Menshchikova T. V., Protogenov A. P. & Chulkov E. V. *et al.* New topological surface state in layered topological insulators: unoccupied Dirac cone. JETP Lett. 96, 780–784 (2012).

[b24] Niesner D. *et al.* Unoccupied topological states on bismuth chalcogenides. Phys. Rev. B 86, 205403 (2012).

[b25] Sobota J. A. *et al.* Direct optical coupling to an unoccupied Dirac surface state in the topological insulator Bi_2_Se_3_. Phys. Rev. Lett. 111, 136802 (2013). 24116801 10.1103/PhysRevLett.111.136802

[b26] Sakai K. Terahertz Optoelectronics (Springer Verlag, 2005).

[b27] Hsieh D. *et al.* A tunable topological insulator in the spin helical Dirac transport regime. Nature 460, 1101–1105 (2009). 19620959 10.1038/nature08234

[b28] Bianchi M. *et al.* Coexistence of the topological state and a two-dimensional electron gas on the surface of Bi_2_Se_3_. Nature Commun. 1, 128 (2010). 21119641 10.1038/ncomms1131

[b29] Urazhdin Bilc S. D., Mahanti S. D. & Tessmer S. H. *et al.* Surface effects in layered semiconductors Bi_2_Se_3_ and Bi_2_Te_3_. Phys. Rev. B 69, 085313 (2004).

[b30] ViolBarbosa C. E. *et al.* Direct observation of band bending in the topological insulator Bi_2_Se_3_. Phys. Rev. B 88, 195128 (2013).

[b31] Galanakis D. & Stanescu D. T. Electrostatic effects and band bending in doped topological insulators. Phys. Rev. B 86, 195311 (2012).

[b32] Ascázubi R., Shneider C., Wilke I., Pino R. & Dutta P. S. *et al.* Enhanced terahertz emission from impurity compensated GaSb. Phys. Rev. B 72, 045328 (2005).

[b33] Sobota J. A. *et al.* Ultrafast electron dynamics in the topological insulator Bi_2_Se_3_ studied by time-resolved photoemission spectroscopy. J. Electron Spectrosc. Relat. Phenom. 195, 249–257 (2014).

