# Supplementary material for: Manifestation of a Second Dirac Surface State and Bulk Bands in THz Radiation from Topological Insulators
Source: Sci Rep. 2015 Sep 15;5:14128. doi: 10.1038/srep14128 (PMC4569898; doi:10.1038/srep14128)
Supplement: Supplementary Information [file srep14128-s1.pdf]

Manifestation of a Second Dirac Surface State and Bulk Bands in THz Radiation from Topological Insulators

Chien-Ming Tu, Tien-Tien Yeh, Wen-Yen Tzeng, Yi-Ru Chen, Hsueh-Ju Chen, Shin-An Ku, Chih-Wei Luo, Jiunn-Yuan Lin, Kaung-Hsiung Wu, Jenh-Yih Juang, Takayoshi Kobayashi, Cheng-Maw Cheng, Ku-Ding Tsuei, Helmuth Berger, Raman Sankar, and Fang-Cheng Chou

Supplementary Information

400-nm excitation on TIs

We performed 400-nm (photon energy E_{photon} = 3.1 eV) excitation experiments to measure P-polarized THz radiation from TIs, and the results are shown in Fig. S1. Comparing the cases of 800-nm and 400-nm excitation, both polarities of THz radiation from n-type $Cu_{0.02}Bi_2Se_3$ and p-type Bi_2Te_3 do not change; meanwhile, a weaker THz radiation from p-type Bi_2Te_3 was observed. In p-type TIs, the direction of diffusion current $\vec{J}_{diffusion}$ is anti-parallel to that of drift current $\vec{J}_{drift}$ [1], as shown in Fig. S2. This means they compete with each other. As the photon energy increases, the diffusion current $\vec{J}_{diffusion}$ will gradually get the upper hand over whole transient current. Finally, the amplitude of the THz radiation from p-type Bi_2Te_3 will suppressed by the appearance of diffusion current $\vec{J}_{diffusion}$ and even the polarity of THz radiation will be reversed again if the photon energy is larger enough and no other effects are considered, as shown in Fig. S2. On the other hand, in n-type TIs, the direction of diffusion current $\vec{J}_{diffusion}$ and drift current $\vec{J}_{drift}$ are the same. Therefore, the amplitude of the THz radiation from n-type $Cu_{0.02}Bi_2Se_3$ will increase as the

photon energy increases. As shown in Fig. S1(a), a little stronger THz radiation from n-type $Cu_{0.02}Bi_2Se_3$ under 400-nm excitation was observed. According to the observation of a weaker THz radiation from p-type Bi_2Te_3 and a little stronger one from n-type $Cu_{0.02}Bi_2Se_3$ under 400-nm excitation, we believe that the surface field effect in TIs is indeed reduced.

In fact, the photon energy (3.1 eV) of 400-nm excitation is much larger than the bandgap (~ 0.3-0.15 eV) of TIs and also larger than the "effective bandgap" (1st band-2nd band). The polarity of THz radiation from p-type Bi_2Te_3 should reverse "again", and it should be the same as that from n-type $Cu_{0.02}Bi_2Se_3$ under 400-nm excitation. However, only the reduction of the amplitude of THz radiation was observed in this study. Comparing with the recent ARPES measurements on Bi_2Se_3 [2], there are other bulk bands around 3 eV and these bulk bands would be responsible for the extra energy loss for 400-nm excitation.

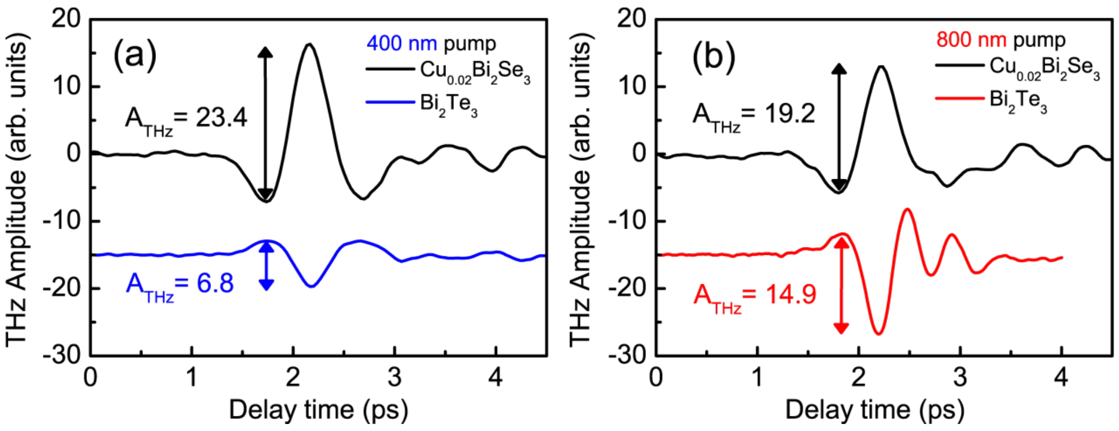

Figure. S1. (a) P-polarized THz radiation from TIs by 400-nm excitation (20 mW). A weaker THz radiation from p-type Bi_2Te_3 is observed. (b) P-polarized THz radiation from TIs by 800-nm excitation (20 mW).

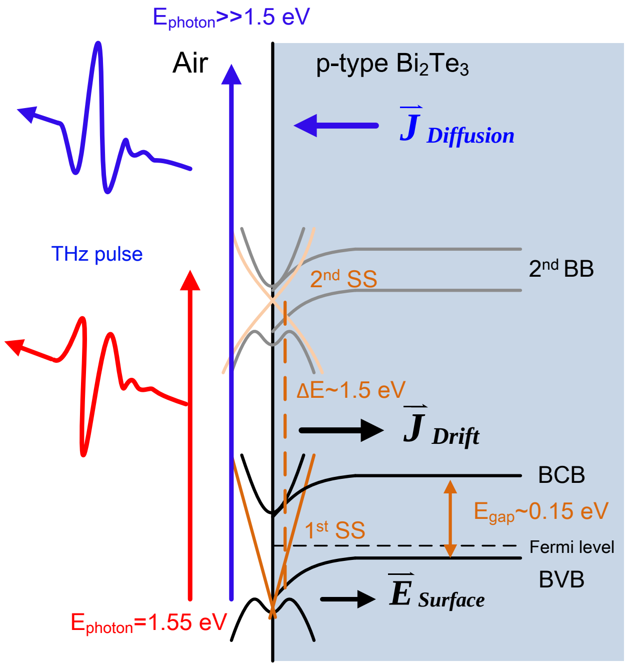

Figure. S2. Schematics of large photon-energy-excitation and THz radiation from p-type Bi_2Te_3.

References

[1] Sakai, K. *Terahertz Optoelectronics* (Springer Verlag, 2005).

[2] Sobota, J. A. *et al*. Direct Optical Coupling to an Unoccupied Dirac Surface State in the Topological Insulator Bi_2Se_3. *Phys. Rev. Lett.* **111**, 136802 (2013).
